# Higher Temperature and Host Age Alter Infection Outcomes in a Multi‐Pathogen System

**DOI:** 10.1002/ece3.74014

**Published:** 2026-07-31

**Authors:** Sarah Elizabeth Troy, Charles E. Mitchell

**Affiliations:** ^1^ The University of North Carolina at Chapel Hill Chapel Hill North Carolina USA

**Keywords:** climate warming, coinfection, host ontogeny, plant pathogen

## Abstract

The outcomes of pathogen infection can be sensitive to temperature, interactions with other pathogen species, and host age. Yet few studies have experimentally tested how warming alters infection outcomes for multiple, potentially interacting, pathogen species across host ages. In this study, we conducted a factorial experiment to test how plant age (7‐week vs. 13‐week plants) and temperature (21°C vs. 29°C) influence infection outcomes of two foliar fungal pathogens with contrasting feeding strategies—*Rhizoctonia solani* (a necrotroph) and *Colletotrichum cereale* (a hemibiotroph)—in the grass species tall fescue ( *Lolium arundinaceum* ). Contrary to expectations of coinfection with pathogens of opposing life‐history strategies leading to increased disease symptoms, coinfection had relatively minor effects across disease metrics. Instead, infection outcomes were driven by host age, pathogen identity and temperature. In plants inoculated with *R. solani* , higher temperature reduced lesion severity, and independently, severity was less in older plants. *C. cereale* lesion development showed a strong age × temperature interaction, with older plants being more resistant to disease in cooler conditions but not under warming. In plants co‐inoculated with both pathogens, elevated temperature reduced disease severity, and this effect was stronger in older plants. These findings demonstrate that environmental conditions and host age can both interact and outweigh within‐host pathogen interactions, highlighting the importance of incorporating host demographic structure in predicting disease responses to climate warming.

## Graphical abstract

Warming, host age, and pathogen identity shaped disease outcomes more strongly than co‐infection in a factorial experiment with two fungal pathogens on tall fescue. Higher temperatures generally reduced disease severity, while host age modified responses‐older plants were more resistant in some contexts, but this depended on temperature and pathogen. These results highlight that host demography and environmental conditions can outweigh within‐host pathogen interactions when predicting disease responses to climate change.

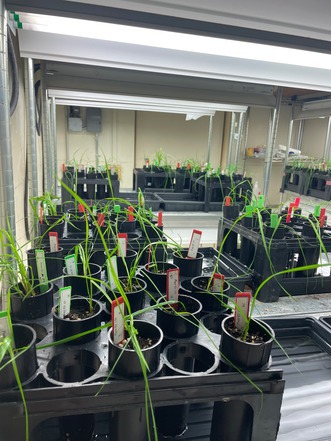

## 1 Introduction

Most free‐living organisms are parasitized by a variety of species or strains over the course of their lives, and can support multiple parasites at one time or in succession with one another (Petney and Andrews 1998; Lord et al. 1999; Cox 2001). Coinfections, where multiple pathogens simultaneously parasitize a single host, are widespread across the tree of life (Glidden et al. 2021; Keen and Dantas 2018; Chun et al. 2024). Exposure of a host to multiple parasites creates the potential for the parasites to ecologically interact within host individuals. Interactions between parasites range from competition, where increases in parasite species richness decreases overall infection success (e.g., Johnson and Hoverman 2012), to facilitation, where infection with one strain can increase host susceptibility to future infections (e.g., Halliday et al. 2020). The role of these interactions in shaping disease transmission within populations is significant (Gorsich et al. 2018; Susi et al. 2015), and in certain contexts, may even override the effects of environmental conditions and host susceptibility (Telfer et al. 2010).

In plants, age and developmental stage play an outsized role in disease susceptibility (Bruns et al. 2022), which is frequently hypothesized to be the result of a trade‐off between growth and defense (Huot et al. 2014; Heckman et al. 2019). Juvenile plants are generally more susceptible to infections, a trend reported in a diverse range of species ranging from potatoes (Gibson 1991) to wheat (Gaudet and Chen 1987). Likewise, abiotic stressors such as drought or heat can further increase susceptibility to disease through a range of molecular and physiological mechanisms, including hormonal cross‐talk, oxidative stress, and repression of defense‐related genes (Zhu 2016; Sewelam et al. 2021; Atkinson and Urwin 2012).

Among abiotic stressors, temperature plays an especially pivotal role, influencing both host physiology and pathogen life‐history. Rising global temperatures and more frequent heat extremes are projected to alter infection outcomes across many plant systems (Velásquez et al. 2018; Garrett et al. 2021). Heat stress can directly compromise host immune responses, modify the timing of defense signaling, and accelerate disease development (Bebber 2015; Prasch and Sonnewald 2013). Because hosts and pathogens often differ in their thermal optima, warming can create divergent responses that shift disease severity and transmission potential (Desaint et al. 2020). These mismatches are particularly consequential for pathogen species interactions within host individuals, where small temperature changes or effects on host physiology can tip competitive or facilitative interactions among coinfecting species (Marçais et al. 2017).

The effects of warming on disease likely depend on host age. Ontogenetic changes in immune signaling, tissue traits, and stress responsiveness can alter how multiple pathogens establish and interact within a single host (Tollenaere et al. 2016; Abdullah et al. 2017; Rejeb et al. 2014). Because age influences both stress signaling and immune capacity, abiotic stress effects on disease are often stage‐specific, with interactions between biotic and abiotic stressors shifting across plant ontogeny (Rejeb et al. 2014; Berens et al. 2019). Despite extensive work on temperature effects on plant disease and separate work on age‐dependent susceptibility, few studies test how warming modifies within‐host pathogen interactions across age groups.

To investigate how interacting biotic and abiotic factors shape disease outcomes, we tested how experimental warming influenced infection dynamics of two fungal pathogens in two age cohorts of vegetative tall fescue ( *Lolium arundinaceum* ) plants. This grass species provides a tractable model for exploring these dynamics, as it commonly hosts multiple fungal pathogens, including *Colletotrichum cereale* and *Rhizoctonia solani* (Halliday et al. 2017). In this study, we conducted a fully factorial controlled grow room experiment that crossed host age (7‐week vs. 13‐week old plants), pathogen inoculation treatment (mock control, single, and co‐inoculations), and temperature (cool vs. heat). This design allowed us to (1) compare disease outcomes between single‐ and multi‐pathogen infections under experimental warming, (2) test how plant age shapes patterns of disease severity, and (3) assess whether warming modifies these age‐dependent dynamics. By linking age and thermal sensitivities, this study experimentally tested how warming can modulate within‐host interactions and thereby potentially reshape patterns of plant disease.

## 2 Methods

### 2.1 Study System

Tall fescue ( *Lolium arundinaceum* ) is a long‐lived, cool‐season perennial grass that ranks among the most economically important forage crops in the United States, covering 30–40 million acres and generating substantial annual profits (Ball et al. 2019; Franzluebbers 1999). Its deep roots and tolerance for a wide range of soils and climates make it a valuable anti‐erosion conservation plant and a dominant species in the Eastern U.S. (Bennett 1979; Buckner et al. 1979; Stephenson et al. 2021). Despite being a cool season grass, tall fescue can persist in transition‐zone states with humid subtropical climates, extending its range into the southern United States (Buckner et al. 1979).

Tall fescue plants experience the most rapid growth in spring and fall, when they form a thicket of upright tillers and a deeply penetrating root system (Buckner et al. 1979). While tall fescue is considered one of the best cool‐season grasses for hot and dry conditions (Turgeon and Vargas 1980), temperatures above 30°C are a key limiting factor of forage production and turf management of tall fescue in the southern United States, as well as in China (Zhang 2005; Hu and Sun 2014). This heat intolerance is more pronounced in younger plants, where seedling growth can be inhibited by temperatures as low as 25°C (Samples et al. 2009). One factor that may partially offset this thermal sensitivity is the widespread association between tall fescue and the systemic, vertically transmitted fungal endosymbiont *Epichloë coenophiala*. This endophyte resides within aboveground plant tissues and is known to enhance host tolerance to abiotic stressors, including heat and drought, as well as herbivory pressure (Xu et al. 2017). In natural and managed populations, where these benefits translate into increased host fitness, *Epichloë* infection frequencies can approach 100%, and it is estimated that 90% of all fescue pastures in the US are *Epichloë*‐infected (Young et al. 2014). Tall fescue is also susceptible to various fungal pathogens, and is known to experience annual disease epidemics which overlap in space, often sharing host populations and individuals, as well as time, with disease epidemics often occurring in tandem over the growing season (Halliday et al. 2017). Two such pathogens are *Rhizoctonia solani*, an aggressive necrotrophic pathogen which rapidly kills leaf tissue, and *Colletotrichum cereale*, a hemi‐biotroph which initially infects living tissue before switching to a necrotic feeding style (Halliday et al. 2018). *R. solani* is a soil‐borne fungus that persists as sclerotia in plant debris and soil and spreads primarily through water‐mediated movement, including splash dispersal from infected soil and residues (Ogoshi 1987). In contrast, *C. cereale* produces conidia, which are dispersed via water splash or direct contact between infected and healthy leaves, facilitating rapid spread within dense foliar canopies (Usman et al. 2026). These pathogens also differ in their disease development within *Lolium arundinaceum* . *R. solani* typically produces water‐soaked lesions that expand rapidly into light brown necrotic patches with distinct darker margins (Ogoshi 1987), reflecting its fast, tissue‐destructive necrotrophic growth strategy. In comparison, *C. cereale* infections begin as small chlorotic lesions that gradually expand into small, dark necrotic patches, often accompanied by the production of acervuli bearing conidia (Usman et al. 2026), consistent with an initial biotrophic phase followed by necrotrophic growth. Both pathogens exhibit temperature‐dependent disease dynamics, though their optimal conditions and field phenology differ. *R. solani* outbreaks are most severe during midsummer heat and are strongly associated with warm, moist conditions that favor rapid mycelial growth and infection (Gross et al. 1998). Similarly, laboratory studies suggest that *C. cereale* can achieve maximal mycelial growth at relatively warm temperatures (~28°C; Wang and Kerns 2017), although field epidemics generally peak in spring (Halliday et al. 2017). While both *R. solani* and *C. cereale* are observed to cause disease across diverse environmental conditions, implying tolerance to a range of temperatures, there is limited direct characterization of their thermal performance in fescue. However, there is evidence from laboratory inoculation experiments that *R. solani* and *C. cereale* benefit from immune‐mediated facilitation, wherein the host immune response to one pathogen suppresses the immune response needed to fend off a pathogen of the opposite feeding guild, resulting in more severe disease symptoms (Green et al. 2024).

Given its overall hardiness paired with age‐dependent sensitivity to elevated temperatures and established coinfection dynamics, tall fescue is an ideal system for examining how warming interacts with host age to shape disease outcomes.

### 2.2 Experimental Overview

#### 2.2.1 Timeline

In this experiment, we established two age cohorts of tall fescue, then inoculated and held them at heated (29°C) or cool (21°C) conditions for 20 days, throughout which they were surveyed to assess disease symptoms and plant growth. Owing to space constraints, only one temperature treatment could be conducted at a time. As a result, the three replicates per temperature treatment were conducted independently, resulting in six total temporal replicates (three per temperature treatment). Each replicate included a full factorial of mock, single, and co‐inoculations across both age groups. Hereafter, “replicate” refers to the temporal replicate of the temperature treatment, with replicates 1, 3, and 5 being the heat treatment at 29°C and 2, 4, and 6 being the cooler treatment at 21°C.

#### 2.2.2 Plant and Fungal Culture Growth

**FIGURE 1:**
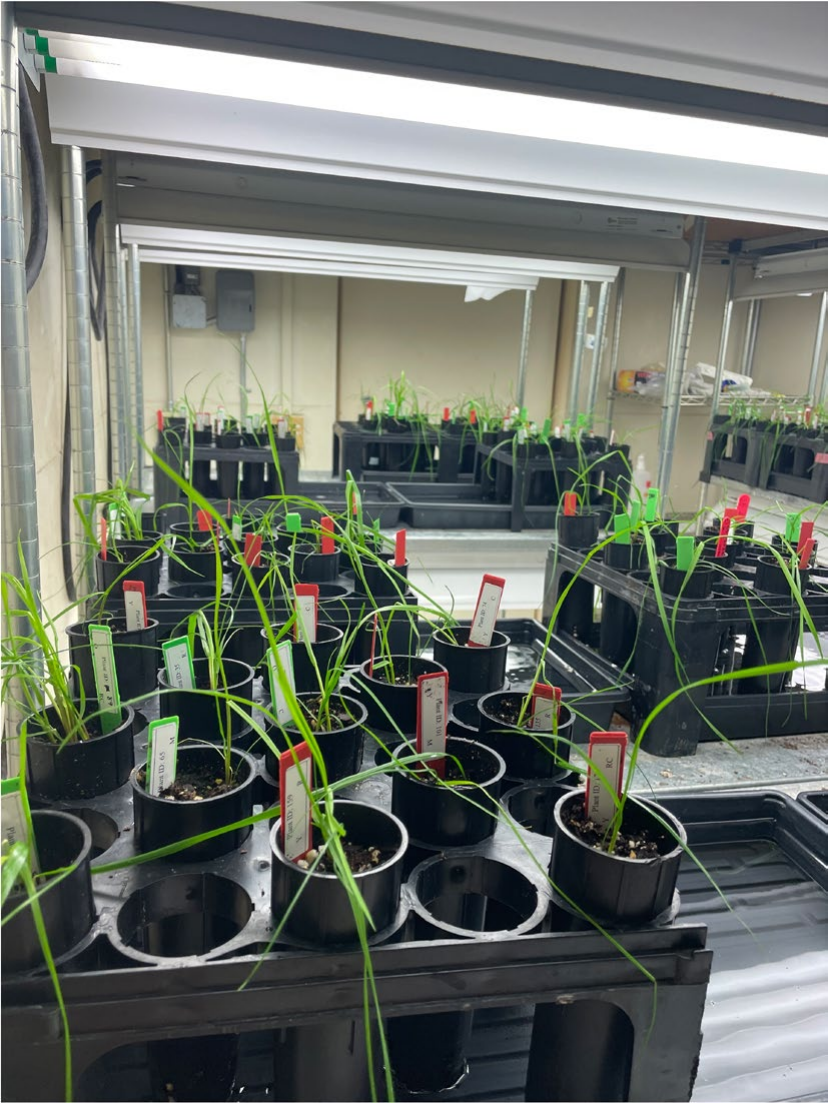
*Lolium arundinaceum* plants in the grow room. Plants were bottom‐watered and kept at 21°C and 20% relative humidity under a 12‐h light/12‐h dark cycle prior to inoculation.

Fescue plants (*N* = 1280) were grown from *Epichloë*‐endophyte‐infected seed stock collected in 2019 from Widener Field (Duke Forest Research and Teaching Laboratory, Orange County, North Carolina, USA), a site characterized by a humid temperate climate with hot summers (mean daily maxima ~31°C) and short, cool winters (National Weather Service 2024). The first cohort (“old plants,” *n* = 640) was planted 13 weeks before inoculation, while the second cohort (“young plants,” *n* = 640) was planted 7 weeks before inoculation. All plants were maintained in the same greenhouse bay at 21°C and 20% relative humidity under a 12‐h light/12‐h dark cycle prior to inoculation, after which they were transferred to a grow room (see below, Figure 1). Cultures of *C. cereale* (6B) and *R. solani* (S1W6) from Widener Field, both originally isolated by F. Halliday and K. O'Keefe in 2015, were grown on potato dextrose agar (PDA) at 3 and 2 weeks prior to inoculation, respectively. These isolates (6B and S1W6) were chosen for their historical success in inoculations done by O'Keeffe et al. (2021), and Grunberg et al. (2023). Germination and culture growth timelines for each replicate are shown in Table 1.

**TABLE 1:** Experimental timeline, inoculation day = 0.

Event	Days relative to inoculation
Old cohort seeds started	−91
Young cohort seeds started	−49
*C. cereale* cultures started	−21
*R. solani* cultures started	−14
Inoculation day	0
Inoculum materials removed	+3
Survey 1	+5
Survey 2	+7
Survey 3	+10
Survey 4	+12
Survey 5	+15
Survey 6	+20

#### 2.2.3 Inoculation and Grow Room Conditions

**FIGURE 2:**
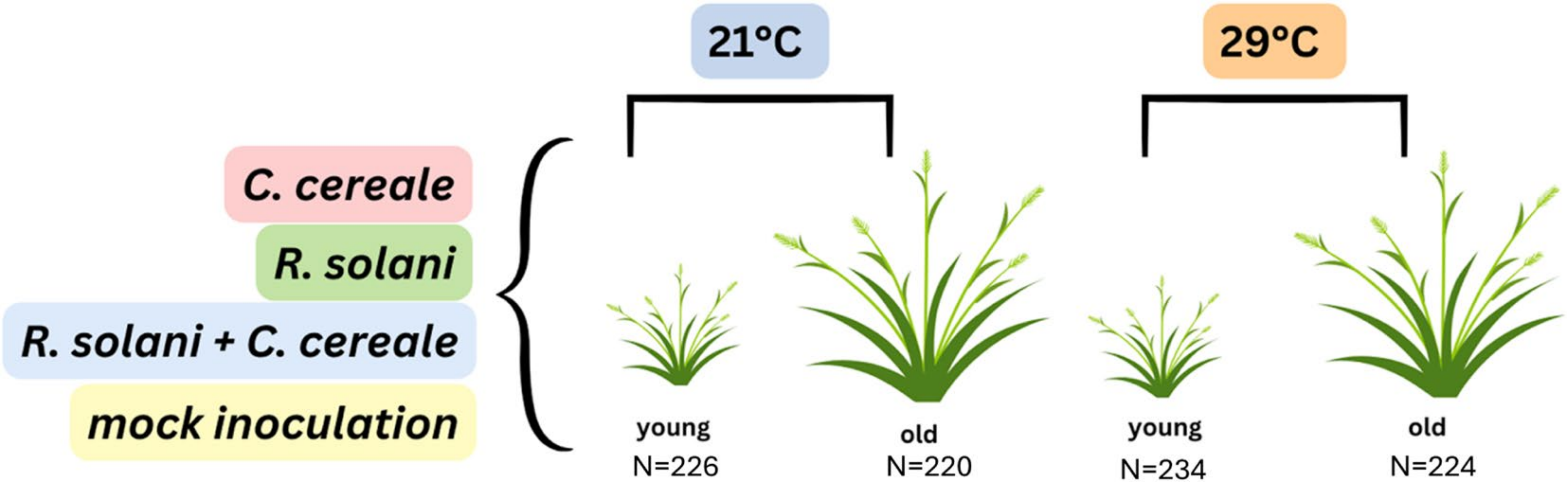
Treatment groups. Within each temperature treatment, old and young *Lolium arundinaceum* plants were assigned to one of four inoculation treatments: Single inoculation with *Colletotrichum cereale* or *Rhizoctonia solani*, co‐inoculation with both pathogens, or a mock inoculation. Singly inoculated plants received a mock treatment for the non‐focal pathogen, and mock‐inoculated plants received two mock inoculations. Sample sizes of surviving plants for each age group × temperature combination are shown.

For each replicate, old and young plants were randomly assigned to one of four groups: (1) Mock‐inoculated plants, which received mock *C. cereale* and *R. solani* inoculations, (2) *R. solani* inoculated plants, which received a *R. solani* inoculation coupled with a mock *C. cereale* inoculation, (3) *C. cereale* inoculated plants, which received a real *C. cereale* inoculation and a mock *R. solani* inoculation, and (4) A co‐inoculation treatment, which received both *R. solani* and *C. cereale* inoculum (Figure 2. *Treatment Groups*). *N* = 25 plants for each inoculum (4) × age group (2) per temperature treatment (2), with the exception of the final replicate (replicate 6, 21°C), which had 35 plants per treatment group. See Table S1 for a detailed breakdown of the sample sizes of individual treatment groups in each temporal replicate.

At day 0 (inoculation day), the youngest leaf > 10 cm was treated with the *C. cereale* or mock *C. cereale* treatment prior to inoculation (or mock inoculation) with *R. solani* . *C. cereale* inoculum was prepared as a spore slurry in potato dextrose broth, with concentrations ranging from 470,100–650,000 conidiospores mL^−1^ (Table S2) as quantified by hemocytometer (Green et al. 2024). The slurry was mixed before each inoculation, and 0.5 mL was painted on each side of the target leaf with a paintbrush. Mock *C. cereale* inoculations were applied using sterile potato dextrose broth. *R. solani* inoculum was applied as a 1‐cm PDA plug of mycelium placed in a foil cup with a moistened cotton pad so the plug contacted the leaf surface (Figure S1). Sham inoculations used plain PDA plugs. After treatment, plants were enclosed in plastic bags for 48 h in the grow room to maintain humidity, after which all inoculum materials were removed. The grow room was set to either 21°C (replicates 2, 4, and 6) or 29°C (replicates 1, 3, and 5) with a 12 h/12 h day–night cycle and maintained at 60%–70% humidity.

#### 2.2.4 Data Collection

Surveys were carried out at 5, 7, 10, 12, 15, and 20 days post‐inoculation. Plants were surveyed one leaf at a time for leaf senescence (death), the presence or absence of any disease symptoms ( *R. solani* —rapidly expanding light brown lesions with a dark brown border, *C. cereale*—chlorotic lesions with a dark brown necrotic center and/or acervuli), disease severity on the inoculated leaf (Percent Leaf Damage), and the total number of tillers (shoots) on the plant. At the end of each replicate, 2 cm segments were collected from the base of the tillers of all surviving plants to test for the presence of *Epichloë coenophiala* using the Agrinostics Phytoscreen Field Tiller Endophyte Detection Kit. *Epichloë* was detected in 98.9% of all plants; to control for any physiological effects caused by its presence, all plants lacking *E. coenophiala* were excluded from further analyses. All remaining aboveground plant tissue was then harvested and dried for biomass measurement.

#### 2.2.5 Data Analysis

All analyses were performed using RStudio (*version 4.4.2, Pile of Leaves*) and each model included random intercepts for treatment blocks nested within replicates to account for variation between blocks. Plant survival was assessed using a generalized linear mixed model (glmer) with a binomial error distribution and logit link (Bates et al. 2015). Survival to the end of the study (number of plants surviving out of the total inoculated) was modeled as a function of the interaction between temperature, age group, and inoculum. Significance of effects was assessed using Type II Wald chi‐square tests, implemented with the Anova() function from the car package (Fox and Weisberg 2019). Only surviving plants were considered in further analyses.

The primary analysis of disease used two metrics—*lesion development* and *disease severity*. Lesion development (whether the plant had developed lesions matching the inoculated pathogen(s) by the final day of observation) was modeled using the same model structure and significance testing as the survival model, with lesion presence at Day 20 as a function of the interactive effects of temperature, age group, and inoculum. For *R. solani* , infection was nearly universal across inoculation treatments, precluding tests of inoculum effects; analyses therefore focused on the effects of temperature and age group.

Disease burden was calculated at each time point by assessing Percent Leaf Damage (hereafter, PLD). PLD was determined by visually assessing the proportion of the inoculated leaf covered in lesions—for example, an inoculated leaf with 50% lesion coverage would receive a PLD score of 0.5. Inoculated leaves which died over the course of the survey period were said to have 100% damage, with a PLD score of 1. The severity of the disease burden experienced by plants over the course of the experiment (*disease severity*) was quantified by calculating the Area Under the Disease Progression Stairs (AUDPS) using the *agricolae* package. AUDPS was defined as the integrated total of PLD over time. To separate disease severity from disease presence/absence, AUDPS was calculated only for plants which developed lesions matching their respective inoculation treatment ( *R. solani* : *n* = 230, *C. cereale*: *n* = 235, coinfection: *n* = 215). Due to overlap between *R. solani* and *C. cereale* infection symptoms (chlorosis, necrosis, and senescence), disease severity measurements for co‐inoculated plants were not differentiated by lesion type. Because the distribution of AUDPS measurements was strongly right‐skewed, values were log‐transformed prior to statistical analysis. Within each inoculum grouping ( *R. solani* , *C. cereale*, or co‐inoculation), log‐transformed AUDPS was modeled as a function of the interactive effects of temperature and age group with a random effect of replicate that was allowed to vary by treatment block.

Differences in dry biomass across temperature, age group, and inoculation treatments were modeled using linear mixed‐effects models, with log‐transformed dry biomass as a function of the interaction between temperature, age group, and inoculum. This model included random intercepts for the treatment blocks nested within replicates to allow for variation between the treatment blocks within each temporal replicate.

## 3 Results

### 3.1 Plant Survival

Out of 1280 plants, 904 (70.6%) survived to the end of the experiment (Day 20) (Appendix A: Table A1). In a generalized linear mixed model with a binomial response and logit link, survival was not significantly affected by temperature (*χ* ^2^_1_ = 0.66, *p* = 0.42) or age group (*χ* ^2^_1_ = 1.09, *p* = 0.30). The main effect of inoculation was not significant (*χ* ^2^_3_ = 3.66, *p* = 0.30), but the two‐way interaction between temperature and inoculation was significant (*χ* ^2^_3_ = 12.17, *p* = 0.007), reflecting variation in survival among inoculum types at different temperatures. At 21°C, survival was highest for plants inoculated with *C. cereale* or *R. solani* (~71%–74%) and lowest for mock and co‐inoculated plants (~59%), while at 29°C, survival was generally higher and more uniform across all inoculum types (71%–81%). All other two and three‐way interactions (temperature × age group, age group × inoculation) were weak and nonsignificant (*χ* ^2^ ≤ 3.06, *p* ≥ 0.38, Appendix A: Table A2).

### 3.2 *C. cereale* Lesion Development

**FIGURE 3:**
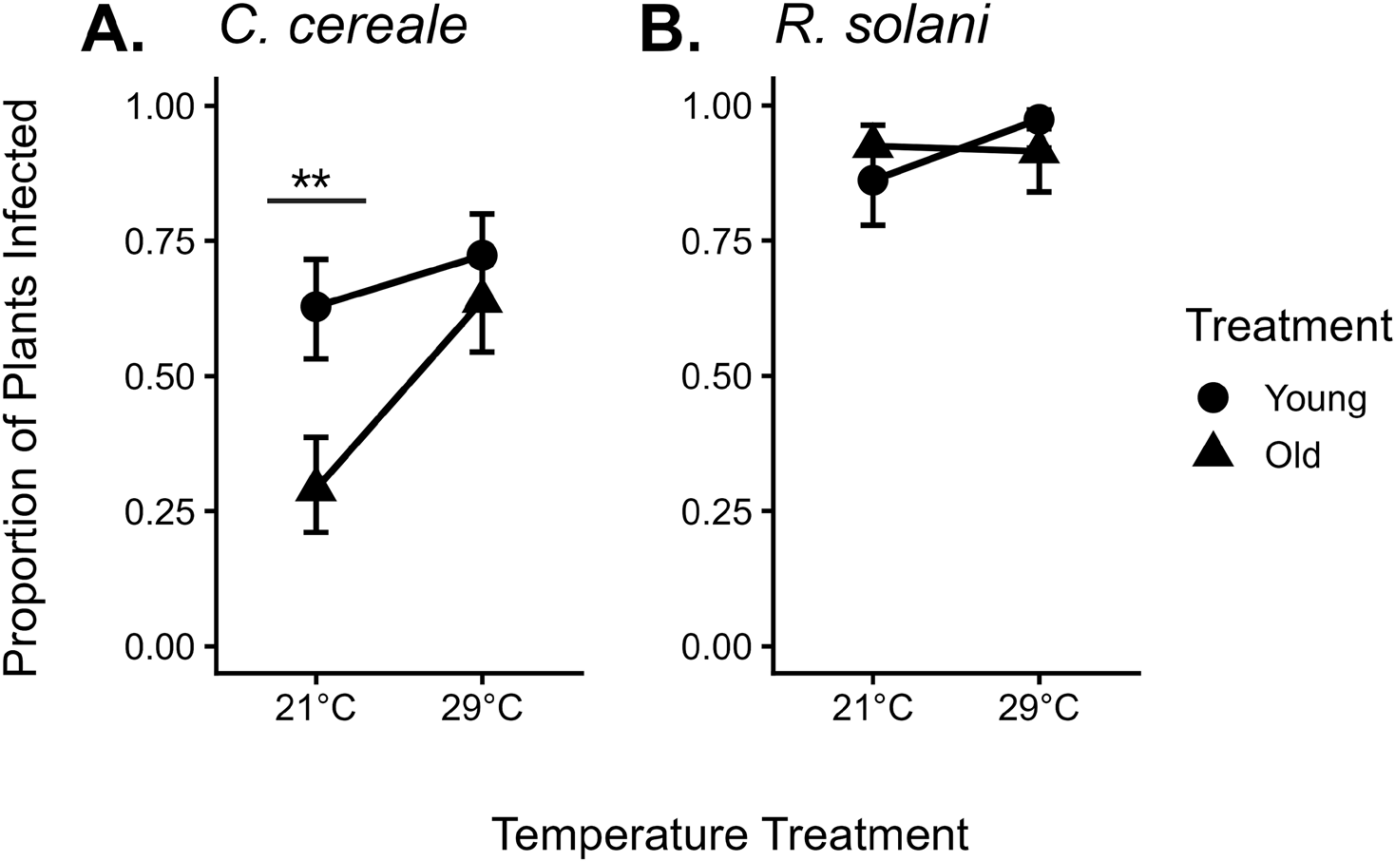
Model‐predicted proportions of *Colletotrichum cereale* (left) and *Rhizoctonia solani* (right) infections/inoculated *Lolium arundinaceum* leaves by age and temperature group. Points show model estimates of the proportion of inoculations resulting in infections (±95% confidence intervals) from a linear mixed‐effects model including temperature, age group, and their interaction (Table A3). Circles represent young plants and triangles represent old plants. At 21°C, younger plants were approximately twice as likely to be infected with *C. cereale* as older plants; whereas at 29°C, there was no significant difference between age groups. There was no significant difference in *R. solani* lesion development between any of the treatment groups (Table A4).

Of plants which survived to the end of the study, 57.8% of singly inoculated plants (136/235) and 56.7% of co‐inoculated plants (122/215) developed *C. cereale* lesions during the survey period. A generalized linear mixed‐effects model (GLMM) with a binomial response and logit link was used to assess what proportion of inoculated plants developed *C. cereale* symptoms by the end of the study (Appendix A: Table A3). Inoculum treatment did not have a significant main effect on lesion development (*χ* ^2^_1_ = 0.34, *p* = 0.56) (Table A3), though plant age and temperature had significant main effects on lesion development, and these effects interacted significantly (Type II Wald *χ* ^2^: age *χ* ^2^_1_ = 17.69, *p* = 2.6E−05; temperature *χ* ^2^_1_ = 18.43, *p* = 1.76E−05; temperature × age *χ* ^2^_1_ = 7.24, *p* = 0.007). Under heat treatment, the amount of lesions at Day 20 was similar between younger and older plants (estimated marginal means: 71% vs. 64%), whereas under cooler conditions, younger plants were more than twice as likely to be infected as older plants (62% vs. 29%) (Figure 3). The effects of inoculum varied with temperature and plant age (temperature × age × inoculum *χ* ^2^₁ = 6.47, *p* = 0.011). This interaction was driven by a strong temperature response in co‐inoculated plants: co‐inoculated plants developed 33% more lesions in the heat treatment compared to cooler conditions, whereas singly inoculated plants showed little differences between temperature treatments (~12%; temperature × inoculum *χ* ^2^_1_ = 5.29, *p* = 0.021).

### 3.3 *R. solani* Lesion Development

Out of 640 plants inoculated with *R. solani* (singly or co‐inoculated with *C. cereale*), 445 survived to the end of the study (Appendix A: Table A1). Observed infection was consistently high, with ~94% of singly inoculated and 90% of co‐inoculated plants developing *R. solani* lesions. Because nearly all plants inoculated with *R. solani* developed lesions, there was insufficient variation in symptom prevalence to meaningfully model the effects of inoculum on symptom development. A generalized linear mixed‐effects model (GLMM) and corresponding Wald Type II Test (Appendix A: Table A4) revealed no statistically significant main effects of temperature or plant age on *R. solani* lesion development by day 20 (*p* > 0.1). There was a significant interaction between temperature and plant age (temperature × age group *χ* ^2^_1_ = 5.59, *p* = 0.018), but the biological magnitude was small, as the proportion of *R. solani* ‐inoculated plants which developed lesions (Figure 3) remained uniformly high across treatments.

### 3.4 Lesion Severity: Area Under the Disease Progression Stairs

Separate linear mixed‐effects models, with treatment nested within replicate temporal blocks as a random effect, were used to test the effects of temperature and plant age on AUDPS of disease severity for *C. cereale*, *R. solani* , and coinfections. Only symptomatic plants whose symptoms matched those of their inoculation type were included in the analyses. Because the symptoms of *R. solani* and *C. cereale* overlap (yellowing, necrosis, senescence), disease severity (percent leaf damage) could not be reliably assigned to either pathogen on coinfected plants, preventing pathogen‐specific comparisons of disease severity via AUDPS.

**FIGURE 4:**
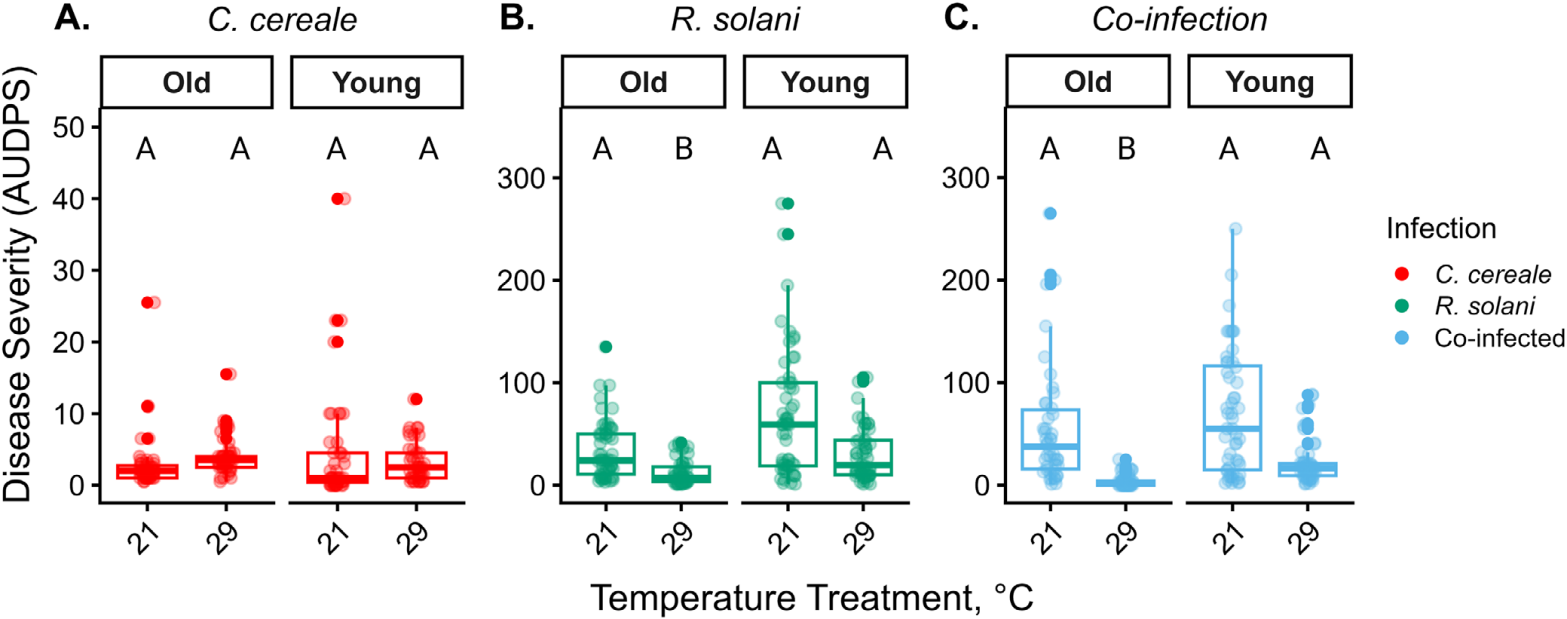
Disease severity (area under the disease progress stairs) responses to temperature and plant age for *Colletotrichum cereale, Rhizoctonia solani*, and coinfected *Lolium arundinaceum* plants. Effects of temperature and age differed among infection types, with additive effects in *R. solani* and strong temperature × age dependence in coinfected plants. No clear treatment effects were observed in *C. cereale*‐infected plants. Letters indicate significant differences among treatment combinations (95% CL of estimated marginal means, *p* < 0.05, Tables A5, A6, A7, A8).

For *C. cereale*, neither age, temperature, nor their interaction were significant predictors of AUDPS (*p* > 0.2; Appendix A: Table A5, Figure 4). In contrast, *R. solani* severity was influenced by independent effects of both temperature and age. Disease burden decreased under the heat treatment (*F*(1,4) = 27.72, *p* = 0.006) and in older plants (*F*(1,4) = 27.31, *p* = 0.006), with no significant interaction between the two (*F*(1,4) = 1.88, *p* = 0.24) (Appendix A: Table A6, Figure 4). The heat treatment suppressed disease severity in both age groups (threefold in older plants and twofold in younger plants), yet younger plants remained consistently (1.7–2.5×) more severely affected than older plants regardless of temperature (estimated marginal means, Appendix A: Table A7).

Overall, the disease severity AUDPS of coinfected plants mirrored those of plants singly infected with *R. solani* , with the heat treatment markedly reducing disease severity, particularly in older plants. However, there was a significant interaction between temperature and age group (temperature: *F*(1,4) = 42.206, *p* = 0.003; age: *F*(1,4) = 47.282, *p* = 0.0023; temperature × age: *F*(1,4) = 31.202, *p* = 0.005) (Appendix A: Table A8, Figure 4). For coinfected plants, the heat treatment reduced disease severity by approximately sixfold in older plants and nearly threefold in younger plants, but younger plants still experienced greater disease burden within temperature treatments (estimated marginal means, Appendix A: Table A9).

### 3.5 Plant Growth

**FIGURE 5:**
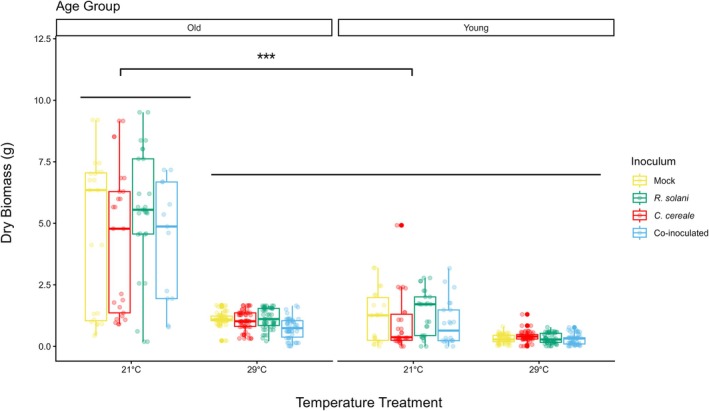
Dry biomass at day 20 postinoculation. Biomass differed across temperature and age treatments, with older plants producing greater biomass overall but experiencing stronger reductions under heat. Inoculum, while statistically significant, had a reduced effect size compared to temperature and age effects. Asterisks indicate significant differences (95% CL of estimated marginal means, *p* < 0.05) among treatment groups. Full statistical results are presented in Table A10.

Dry biomass at the end of the experiment was strongly influenced by both plant age and temperature. As expected, given their 6 weeks of additional growth prior to inoculation, older plants had nearly twice the biomass of younger plants (*F*(1,28) = 457.9, *p* < 0.0001; Estimate = +0.823; Appendix A: Table A10). Heat treatment reduced biomass by approximately one‐third overall (*F*(1,4) = 461.7, *p* < 0.0001; Estimate = −0.404), but this effect differed by age group (age × temperature: *F*(1,28) = 80.8, *p* < 0.0001). Biomass in older plants declined sharply under heat, from 4.82 g at 21°C to 1.95 g at 29°C, whereas younger plants showed a more modest decline, from 1.91 g to 1.30 g (estimated marginal means; Appendix A: Table A11). Inoculum had a statistically detectable effect on biomass (*F*(3,28) = 6.868, *p* = 0.0013) and a weak interaction with temperature (*F*(3,28) = 3.31, *p* = 0.034), but these effects were minor relative to the strong and consistent influences of age and temperature, and no significant interactions involving age were detected (Table A10; Figure 5).

## 4 Discussion

In this controlled warming experiment, we tested how host age and temperature influence infection outcomes in a multi‐pathogen system. Contrary to expectations of immune‐mediated facilitation between fungal pathogens of contrasting feeding guilds, coinfection effects were minimal across most metrics. Instead, disease dynamics were structured primarily by pathogen identity and the interaction between host age and temperature, highlighting how environmental (Velásquez et al. 2018) and ontogenetic (Develey‐Rivière and Galiana 2007) effects can outweigh within‐host pathogen interactions.

### 4.1 Limited Evidence for Facilitation in Coinfections

Previous work has proposed that *R. solani* and *C. cereale* may facilitate one another through antagonistic immune signaling pathways (Halliday et al. 2018; O'Keeffe et al. 2021; Green et al. 2024), resulting in greater disease burden as a result of salicylic acid and jasmonic acid‐mediated tradeoffs (Thaler et al. 2012; Glazebrook 2005; Vlot et al. 2009). However, we found little evidence that co‐inoculation altered lesion development, cumulative disease severity, plant survival, or final biomass relative to single infections. Because of overlap in the symptoms of *R. solani* and *C. cereale* (yellowing, necrosis, senescence), damage to coinfected leaves could not be confidently partitioned by pathogen, preventing pathogen‐specific assessments of lesion severity and reducing our ability to detect shifts in competitive dominance under coinfection. Nonetheless, cumulative disease damage (AUDPS) in co‐inoculated plants closely resembled that of *R. solani* single infections, suggesting that *R. solani* was the primary driver of lesion expansion in coinfections.

Several factors may explain the weak coinfection signal. Simultaneous—rather than sequential—inoculation may have limited the opportunity for immune modulation to influence subsequent disease establishment (Green et al. 2024; Clark et al. 2013). Additionally, the near‐ubiquitous success of *R. solani* likely reduced variation in early infection dynamics, constraining our ability to detect facilitative or competitive shifts during the establishment phase. More broadly, the strong influence of temperature and host age across treatments suggests that demographic and abiotic filters may define the conditions under which pathogen interactions meaningfully influence disease outcomes.

#### 4.1.1 Infection Establishment and Disease Progression Respond to Different Drivers

Infection establishment and lesion expansion responded to distinct combinations of host age and temperature, demonstrating how “disease severity” comprises multiple components that do not necessarily respond uniformly to environmental change (Seem 1984). A potential unifying mechanism underlying these patterns may be age‐ and temperature‐associated shifts in leaf tissue physiology, particularly structural and cell wall–associated defenses. Across ontogeny, leaves often exhibit changes in cell wall composition, thickness, and reinforcement, contributing to age‐based resistance (Alzohairy et al. 2023). Temperature can further modify these traits by altering leaf thickness, cell wall architecture, and structural investment (Schollert et al. 2015). One well‐characterized component of this structural remodeling is lignification—the deposition of lignin and callose within the cell wall—which reinforces and hardens plant tissues and is frequently induced by biotic or abiotic stressors (Wang et al. 2013). Increased lignin deposition can reduce pathogen penetration efficiency and limit tissue digestibility (Vance et al. 1980; Scott‐Craig et al. 1995). For example, enhanced lignification has been associated with reduced disease progression in tolerant olive cultivars infected with *Xylella fastidiosa* (Sabella et al. 2018) and with decreased late blight severity in potato (Purwantisari et al. 2020).

For *R. solani* , infection establishment was largely insensitive to host age or temperature, with nearly all inoculated plants developing lesions. However, disease severity was moderated by both factors, with older plants and those in the heat treatment consistently showing less cumulative damage. In this case, warming did not influence whether infection occurred, but instead constrained lesion expansion after establishment. This pattern is consistent with structural resistance limiting pathogen spread (Lee et al. 2019), and suggests that host age and warming may reduce post‐establishment progression by modifying tissue quality or reinforcing cell wall–associated defenses.

Overall, the heat treatment resulted in increased *C. cereale* lesion development, and the trend was more pronounced in co‐inoculated plants. *C. cereale* also exhibited a strong age × temperature interaction during lesion development. Under cool conditions, younger plants were substantially more likely to develop lesions than older plants, reflecting pronounced age‐dependent susceptibility during infection establishment, consistent with known effects of plant age on immune recognition and early defense deployment (Huot et al. 2014). Older plants also typically possess greater structural reinforcement, which can limit appressorial penetration and reduce infection probability (Koeck et al. 2011; Tiwari et al. 2025). However, under heated conditions, this age‐based difference narrowed considerably, potentially due to increased pathogen growth and enzymatic activity or temperature‐induced suppression of early immune signaling (Koeck et al. 2011; Zhu 2016; Sewelam et al. 2021; Atkinson and Urwin 2012). Once *C. cereale* transitions to necrotrophy, hyphae spread from previously infected cells into neighboring tissue, a process which bypasses some of the structural and immune barriers faced during initial infection (Usman et al. 2026).

#### 4.1.2 Warming Reduced Age‐Based Differences in Disease Symptoms

A notable emergent pattern was the reduction of age‐based heterogeneity in disease expression under warming. For *C. cereale*, warming diminished differences in infection establishment between young and old plants. For *R. solani* , warming equalized cumulative severity (AUDPS) across age classes. In both cases, elevated temperature shifted disease outcomes toward greater uniformity among hosts. This convergence of disease symptoms suggests that elevated temperatures can partially override age‐based differences in plant susceptibility, potentially by shifting host physiology toward a uniform stress response. Rather than universally amplifying or suppressing disease, warming may therefore restructure the distribution of susceptibility within age‐structured plant populations.

### 4.2 Growth and Performance Under Infection

Biomass responses were dominated by an interaction between age and temperature, with older plants experiencing substantial growth reductions under heat. Despite differences in lesion development and cumulative damage, inoculation treatments had relatively minor effects on final biomass and survival within temperature‐age groups. However, the detection of measurable disease effects over only a 20‐day survey period is itself notable, particularly given that inoculations were restricted to a single leaf per plant. While both pathogens were inoculated under highly favorable conditions for infection, these results nevertheless demonstrate that pathogen impacts can emerge rapidly and be detectable even over short timescales. At the same time, the limited whole‐plant performance costs observed here may reflect the short experimental duration or localized nature of inoculation. Longer‐term experiments may reveal delayed or cumulative performance costs not captured here. Additionally, measurements of photosynthetic performance or carbon and nitrogen concentrations could help clarify whether leaf‐level pathology translated into physiological costs not yet reflected in whole‐plant growth.

### 4.3 Implications for Disease Dynamics Under Environmental Change

Our findings demonstrate that host age and temperature interact in pathogen‐specific and stage‐dependent ways, and support that environmental change may restructure disease processes rather than uniformly amplifying or suppressing severity. Within‐host pathogen interactions were weaker than expected, emphasizing that demographic structure and abiotic context can overshadow coinfection effects. In age‐structured populations, warming‐induced reductions in the distribution of susceptibility between age classes could alter epidemic trajectories in nonintuitive ways. Moreover, the pathogen‐specific responses observed here indicate that warming may restructure pathogen communities by differentially influencing establishment versus progression depending on life history strategy.

By integrating host ontogeny with experimental warming in a multi‐pathogen framework, this study highlights the importance of demographic structure in forecasting disease responses to environmental change. Within tall fescue systems, these dynamics could have direct functional consequences: shifts in age structure and pathogen dominance under warming may alter the seasonal timing and intensity of disease, potentially affecting forage yield and quality in pasture systems and for turf density, esthetics, and persistence in managed landscapes. Future work linking within‐host physiological shifts to transmission processes in field settings will be critical for predicting how climate warming reshapes disease risk within plant populations.

## Author Contributions

**Sarah Elizabeth Troy:** conceptualization (lead), data curation (lead), formal analysis (lead), investigation (lead), methodology (lead), project administration (lead), resources (equal), visualization (equal), writing – original draft (lead), writing – review and editing (equal). **Charles E. Mitchell:** conceptualization (supporting), data curation (supporting), formal analysis (supporting), funding acquisition (lead), methodology (supporting), resources (equal), supervision (equal), visualization (supporting), writing – review and editing (equal).

## Funding

This work was supported by the National Institute of Food and Agriculture, 2016‐67013‐25762. National Science Foundation, DEB‐2308472.

## Conflicts of Interest

The authors declare no conflicts of interest.

## Supporting information


**Figure S1:** ece374014‐sup‐0001‐FigureS1.png. *Rhizoctonia solani* inoculum application. The youngest leaf longer than 10 cm (bracketed) was selected for inoculation. PDA plugs (⅛″) were placed on the leaf surface and gently wrapped with a 3 × 3 in. piece of aluminum foil containing a sterile water‐soaked cotton pad.

**Table S1:** Sample size and plant survival by treatment group and temporal replicate.
**Table S2:** ece374014‐sup‐0002‐Tables.docx. *C. cereale* spore concentration in each replicate.

## Data Availability

The data and code which support the findings of this study are openly available in the Dryad digital repository at DOI: 10.5061/dryad.h1893202b.

## Appendix A

**TABLE A1:** Total number of plants inoculated and survived to day 20 by treatment group.

Treatment group	Group	*N*(inoculated)	*N*(survived)
Temporal replicate	1	200	153
	2	200	98
	3	200	153
	4	200	97
	5	200	152
	6	280	251
Temperature (°C)	21	680	446
	29	600	458
Age group	Old	640	444
	Young	640	460
Inoculum	Mock	320	224
	*C. cereale*	320	235
	*R. solani*	320	230
	Co‐inoculation	320	215

**TABLE A2:** Type II Wald chi‐square tests for a generalized linear mixed model of plant survival across treatment groups.

Term	NumDF	*χ* ^2^	Pr(> *χ* ^2^)	Significance
Temperature	1	0.6613	0.4161	NS
Age group	1	1.0935	0.2957	NS
Inoculum	3	3.6598	0.3006	NS
Temperature × age group	1	0.0735	0.7863	NS
Temperature × inoculum	3	12.1679	0.0068	**
Age group × inoculum	3	3.0586	0.3827	NS
Temperature × age group × inoculum	3	0.2821	0.9634	NS

*Note:* *N*(plants) = 904. glmm: (survived/died)~temperature treatment (21°C or 29°C) × age group (young or old) × Inoculum (single or co‐inoculation) + (1|replicate/treatment block). NS, not significant (*p* ≥ 0.05); ***p* < 0.01.

**TABLE A3:** Type II Wald chi‐square test for a generalized linear mixed model of the effects of inoculum, temperature, and plant age on *C. cereale* lesion development at day 20 postinoculation.

Term	NumDF	*χ* ^2^	Pr(> *χ* ^2^)	Significance
Temperature	1	12.7192	0.0004	***
Age group	1	10.5597	0.0012	**
Inoculum	1	0.3442	0.5574	NS
Temperature × age group	3	3.9459	0.05	*
Temperature × inoculum	3	5.2947	0.0214	*
Age group × inoculum	3	0.2865	0.5924	NS
Temperature × age group × inoculum	3	6.4697	0.011	*

*Note:* *N*(plants) = 445; *n*(single Inoculation) = 230, *n*(co‐inoculation) = 215. glmm: (infected, total‐infected)~temperature treatment (21°C or 29°C) × age group (young or old) × Inoculum (single or co‐inoculation) + (1|replicate/treatment block). NS, not significant (*p* ≥ 0.05); **p* < 0.05; ***p* < 0.01; ****p* < 0.001.

**TABLE A4:** Type II Wald chi‐square test for a generalized linear mixed model of the effects of temperature and plant age on *R. solani* lesion development at day 20 postinoculation.

Term	NumDF	*χ* ^2^	Pr(> *χ* ^2^)	Significance
Temperature	1	1.956	0.162	NS
Age group	1	0.049	0.825	NS
Temperature × age group	3	5.586	0.0181	*

*Note:* *N*(plants) = 445; *n*(single Inoculation) = 230, *n*(co‐inoculation) = 215. glmm: (infected, total‐infected)~temperature treatment (21°C or 29°C) × age group (young or old) + (1|replicate/treatment block). NS, not significant (*p* ≥ 0.05); **p* < 0.05.

**TABLE A5:** ANOVA posthoc test of linear mixed effects model of the effects of temperature and age group on log‐transformed AUDPS in plants singly infected with *C. cereale*.

Term	NumDF	DenDF	*F*‐value	*p*	Significance
(Intercept)	1	177	198.348	< 0.0001	***
Temperature	1	4	0.529	0.507	NS
Age group	1	4	0.343	0.589	NS
Temperature × age group	1	4	1.664	0.267	NS

*Note:* Model: log‐transformed AUDPS~temperature (21°C or 29°C) × age group (young or old) + (1 | replicate/treatment block). *N* = 178. NS, not significant (*p* ≥ 0.05); ****p* < 0.001.

**TABLE A6:** ANOVA posthoc test of linear mixed‐effects model of the effects of temperature and age group on log‐transformed AUDPS in plants singly infected with *R. solani* .

Term	NumDF	DenDF	*F*‐value	*p*	Significance
(Intercept)	1	216	1456.343	< 0.0001	***
Temperature	1	4	27.7154	0.0062	**
Age group	1	4	27.3054	0.0064	**
Temperature × age group	1	4	1.8811	0.2421	NS

*Note:* Model: log‐transformed AUDPS~temperature (21°C or 29°C) × age group (young or old) + (1 | replicate/treatment block). *N* = 216. NS, not significant (*p* ≥ 0.05); ***p* < 0.01; ****p* < 0.001.

**TABLE A7:** Estimated marginal means from LMM for log AUDPS of *R. solani* ‐infected plants.

Temperature	Age group	Emmean (AUDPS)	SE	df	Lower.CL	Upper.CL
21°C	Old	25.03	3.68	4	16.63	37.73
21°C	Young	42.52	6.25	4	28.23	64.29
29°C	Old	8.76	1.34	4	5.70	13.32
29°C	Young	21.54	3.19	4	14.29	32.50

*Note:* EMM, Standard errors, and 95% Confidence Limits are back‐transformed from log AUDPS.

**TABLE A8:** ANOVA posthoc test of linear mixed effects model of the effects of temperature and age group on log‐transformed AUDPS in plants coinfected with *C. cereale* and *R. solani* .

Term	NumDF	DenDF	*F*‐value	*p*	Significance
(Intercept)	1	198	417.596	< 0.0001	***
Temperature	1	4	42.206	0.0029	**
Age group	1	4	47.282	0.0023	**
Temperature × age group	1	4	31.202	0.005	**

*Note:* Model: log‐transformed AUDPS~temperature (21°C or 29°C) × age group (young or old) + (1 | replicate/treatment block). *N* = 198. ***p* < 0.01; ****p* < 0.001.

**TABLE A9:** Estimated marginal means from LMM for log AUDPS of coinfected plants.

Temperature	Age group	Emmean (AUDPS)	SE	df	Lower.CL	Upper.CL
21°C	Old	38.599	1.256	4	20.482	72.740
21°C	Young	43.863	1.246	4	23.801	80.836
29°C	Old	2.815	1.241	4	1.544	5.131
29°C	Young	16.202	1.237	4	8.967	29.272

*Note:* EMM, Standard errors, and 95% Confidence Limits are back‐transformed from log AUDPS.

**TABLE A10:** ANOVA posthoc test of linear mixed‐effects model of log dry biomass as a product of inoculum, temperature treatment, and age group.

Term	NumDF	DenDF	*F*‐value	*p*	Significance
(Intercept)	1	899	2421.8911	< 0.0001	***
Temperature	1	4	461.7309	< 0.0001	***
Age group	1	28	457.9135	< 0.0001	***
Inoculum	3	28	6.8676	0.0013	**
Temperature × age group	1	28	80.8014	< 0.0001	***
Temperature × inoculum	3	28	3.3143	0.0343	*
Age group × inoculum	3	28	1.1319	0.3531	NS
Temperature × age group × inoculum	3	28	1.2082	0.3250	NS

*Note:* Model: log(dry biomass)~Inoculum (single or co‐inoculation) × age group (young or old) × temperature treatment (21°C or 29°C) × age group (young or old) × Inoculum (single or co‐inoculation) + (1|replicate/treatment block). NS, not significant (*p* ≥ 0.05); **p* < 0.05; ***p* < 0.01; ****p* < 0.001.

**TABLE A11:** Estimated marginal means from LMM for log dry biomass.

Temperature	Age group	Emmean (g)	SE	df	Lower.CL	Upper.CL
21°C	Old	4.82	0.18	28	4.39	5.30
21°C	Young	1.91	0.06	28	1.75	2.08
29°C	Old	1.95	0.04	28	1.84	2.08
29°C	Young	1.30	0.03	28	1.23	1.38

*Note:* EMM, Standard errors, and 95% Confidence Limits are back‐transformed from log dry biomass.
