# Supplementary figures and images for: Higher Temperature and Host Age Alter Infection Outcomes in a Multi‐Pathogen System
Source: Ecol Evol. 2026 Jul 31;16(8):e74014. doi: 10.1002/ece3.74014 (PMC13426325; doi:10.1002/ece3.74014)

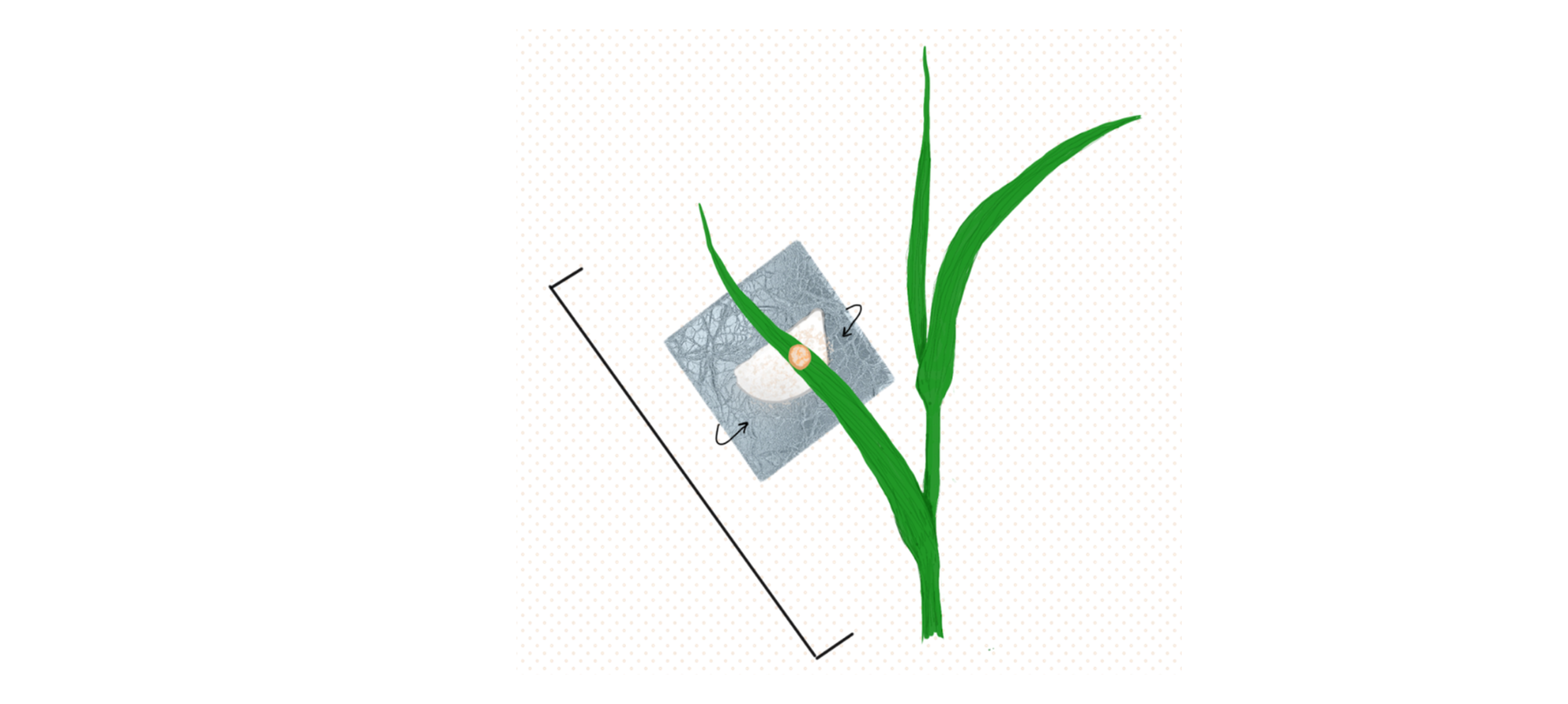

Supplement: Supplementary file 1 — Figure S1: ece374014‐sup‐0001‐FigureS1.png. Rhizoctonia solani inoculum application. The youngest leaf longer than 10 cm (bracketed) was selected for inoculation. PDA plugs (⅛″) were placed on the leaf surface and gently wrapped with a 3 × 3 in. piece of aluminum foil containing a sterile water‐soaked cotton pad. [file ECE3-16-e74014-s002.png]
