# Supplementary material for: Higher Temperature and Host Age Alter Infection Outcomes in a Multi‐Pathogen System
Source: Ecol Evol. 2026 Jul 31;16(8):e74014. doi: 10.1002/ece3.74014 (PMC13426325; doi:10.1002/ece3.74014)
Supplement: Supplementary file 2 — Table S1: Sample size and plant survival by treatment group and temporal replicate. Table S2: ece374014‐sup‐0002‐Tables.docx. C. cereale spore concentration in each replicate. [file ECE3-16-e74014-s001.docx]

| **Table S1. Sample size and plant survival by treatment group and temporal replicate.** | | | | | |
| --- | --- | --- | --- | --- | --- |
| **Temporal Replicate** | **Temperature (℃)** | **Age Group** | **Inoculum** | **N(inoculated)** | **N(Survived)** |
| 1 | 29 | Old | *C. cereale* | 25 | 19 |
| 1 | 29 | Old | Mock | 25 | 21 |
| 1 | 29 | Old | *R. solani* | 25 | 17 |
| 1 | 29 | Old | co-inoculation | 25 | 18 |
| 1 | 29 | Young | *C. cereale* | 25 | 20 |
| 1 | 29 | Young | Mock | 25 | 20 |
| 1 | 29 | Young | *R. solani* | 25 | 18 |
| 1 | 29 | Young | co-inoculation | 25 | 20 |
| 2 | 21 | Old | *C. cereale* | 25 | 13 |
| 2 | 21 | Old | Mock | 25 | 10 |
| 2 | 21 | Old | *R. solani* | 25 | 15 |
| 2 | 21 | Old | co-inoculation | 25 | 9 |
| 2 | 21 | Young | *C. cereale* | 25 | 13 |
| 2 | 21 | Young | Mock | 25 | 10 |
| 2 | 21 | Young | *R. solani* | 25 | 13 |
| 2 | 21 | Young | co-inoculation | 25 | 15 |
| 3 | 29 | Old | *C. cereale* | 25 | 19 |
| 3 | 29 | Old | Mock | 25 | 20 |
| 3 | 29 | Old | *R. solani* | 25 | 17 |
| 3 | 29 | Old | co-inoculation | 25 | 18 |
| 3 | 29 | Young | *C. cereale* | 25 | 20 |
| 3 | 29 | Young | Mock | 25 | 20 |
| 3 | 29 | Young | *R. solani* | 25 | 19 |
| 3 | 29 | Young | co-inoculation | 25 | 20 |
| 4 | 21 | Old | *C. cereale* | 25 | 14 |
| 4 | 21 | Old | Mock | 25 | 9 |
| 4 | 21 | Old | *R. solani* | 25 | 13 |
| 4 | 21 | Old | co-inoculation | 25 | 11 |
| 4 | 21 | Young | *C. cereale* | 25 | 14 |
| 4 | 21 | Young | Mock | 25 | 9 |
| 4 | 21 | Young | *R. solani* | 25 | 14 |
| 4 | 21 | Young | co-inoculation | 25 | 13 |
| 5 | 29 | Old | *C. cereale* | 25 | 19 |
| 5 | 29 | Old | Mock | 25 | 21 |
| 5 | 29 | Old | *R. solani* | 25 | 17 |
| 5 | 29 | Old | co-inoculation | 25 | 18 |
| 5 | 29 | Young | *C. cereale* | 25 | 19 |
| 5 | 29 | Young | Mock | 25 | 20 |
| 5 | 29 | Young | *R. solani* | 25 | 19 |
| 5 | 29 | Young | co-inoculation | 25 | 19 |
| 6 | 21 | Old | *C. cereale* | 35 | 32 |
| 6 | 21 | Old | Mock | 35 | 34 |
| 6 | 21 | Old | *R. solani* | 35 | 33 |
| 6 | 21 | Old | co-inoculation | 35 | 27 |
| 6 | 21 | Young | *C. cereale* | 35 | 33 |
| 6 | 21 | Young | Mock | 35 | 30 |
| 6 | 21 | Young | *R. solani* | 35 | 35 |
| 6 | 21 | Young | co-inoculation | 35 | 27 |
|  |  |  |  | N= 1,280 | N= 904 |

| **Table S2. *C. cereale* spore concentration in each replicate** | |
| --- | --- |
| **Replicate** | **Spore concentration (conidiospores/ml^-1)** |
| 1 | 650,000 |
| 2 | 541,400 |
| 3 | 600,200 |
| 4 | 470,100 |
| 5 | 525,700 |
| 6 | 610,800 |
